# Editorial: Emotional regulation and human flourishing: theoretical and empirical perspectives

**DOI:** 10.3389/fpsyg.2025.1565130

**Published:** 2025-03-19

**Authors:** Pía Valenzuela, Claudia Navarini, Juan A. Mercado, Blaine Fowers, Angelo Panno

**Affiliations:** ^1^Faculty of Philosophy, Pontificia Università della Santa Croce, Rome, Italy; ^2^Department of Health and Life Sciences, Università Europea di Roma, Rome, Italy; ^3^Department of Educational and Psychological Studies, University of Miami, Coral Gables, FL, United States

**Keywords:** emotions, reason, regulation, flourishing, meaning, action, motive, virtue

The relationship between emotional regulation and human flourishing has emerged as a vital area of interdisciplinary inquiry, bridging philosophy, psychology, and education (Gross, 1999, 2015; Thompson, 1994; Tamir, 2016; Kristjánsson, 2018, 2019; Mercado, 2020; Mercado and Valenzuela, 2020). Historically, emotions have often been perceived as obstacles to reason and social harmony, a viewpoint that has shaped much of Western thought (Scheler, 1973; Arnold, 1960; Goldie, 2000). However, contemporary research reveals that emotions are deeply intertwined with cognition and morality, playing a crucial role in shaping our perceptions, memories, and moral judgments (Steinbock, 2014; De Monticelli, 2018). Far from being mere disruptors, emotions guide human behavior in ways that enable effective responses to life's circumstances (Fredrickson, 2018; Navarini, 2023). Emotions serve a variety of functions such as facilitating communication, guiding decision-making, and promoting social bonding (Keltner and Haidt, 2001; Tyng et al., 2017; Šimić et al., 2021). For instance, anger heightens sensitivity to injustice, prompting actions that seek to restore fairness (Ekman, 1999), while emotions such as love and sympathy underpin the complex social bonds that define human relationships (Arnold, 1960).

This Research Topic of articles builds upon these insights by exploring the mechanisms and strategies that allow individuals to navigate their emotional landscapes in ways that promote flourishing. By integrating perspectives from psychology, philosophy, and beyond, the contributions delve into the cognitive, affective, and ethical dimensions of emotion regulation, positioning it as a cornerstone of wellbeing (Fowers et al., 2021, 2024). Through this lens, the research examines how individuals regulate emotions not only to feel better but also to align their responses with meaningful life goals and societal values (Panno et al., 2013; Mercado, 2021).

A recurring theme in these studies is the call for a broader understanding of emotional experiences and/or the construct of flourishing. The work of De Jesús Gómez and Cornu-Labat challenges conventional constructs such as emotional intelligence, advocating instead for the concept of “affectivity,” which captures the full spectrum of all affective experiences and not only the emotional ones. This perspective invites researchers to rethink the scope of emotional regulation, suggesting the term “affective regulation”, which emphasizes its integrative potential in shaping individual realities.

Similarly, Martínez-Priego et al. conduct the complex of systematically reviewing models of emotion regulation, juxtaposing contemporary frameworks with classical philosophical insights. Their valuable analysis highlights a critical gap in existing approaches: the lack of integration between hedonic goals, which prioritize pleasure, and eudaimonic aspirations, which focus on growth and meaning. Revisiting the insights of Aristotle, Descartes and Darwin, the authors underscore the need for models that balance these dimensions, fostering a deeper understanding of flourishing as a multidimensional construct.

The interplay between motivation and emotional regulation is examined by Curren and Park through the lens of Self-Determination Theory. Their work critiques the limitations of goal-directed models, advocating for an approach that aligns emotional regulation with self-determination and eudaimonic functioning. This perspective not only broadens the scope of emotion regulation research but also underscores its relevance to human flourishing.

The theoretical implications of emotional regulation extend to practical applications in the work of Ruiz-Fuster et al. Drawing on Magda Arnold's concept of the “self-ideal,” they propose a framework that links emotional regulation with constructive life goals, and thus, motivation, providing a pathway for individuals to align their emotional responses with their aspirations. Emotional regulation can be fostered by meaningful goals, which facilitate, in turn, a flourishing life. This holistic approach encompasses all dimensions of wellbeing, integrating psychological and eudaimonic aspects while also providing valuable insights for interventions addressing emotion dysregulation.

A different perspective on flourishing comes from Novak and Kiknadze, who critically examine the role of positive and negative emotions in defining the “good life.” Their work challenges the privileging of positive emotions in many flourishing models, arguing for a more nuanced understanding that respects cultural diversity and individual differences. By advocating for balance and complexity, their analysis invites researchers to reconsider simplistic notions of wellbeing.

Innovative approaches to emotion regulation are also evident in the therapeutic domain. Hauke et al. integrate embodiment into Cognitive Behavioral Therapy, emphasizing the bidirectional relationship between body and psyche. By incorporating physical sensations and movements, their approach enhances emotional meaning-making, offering new pathways for therapeutic interventions. This embodied perspective highlights the potential of emotion regulation techniques to address prelinguistic and hard-to-access emotional experiences.

The complexities of emotion dysregulation, particularly during adolescence, are explored by Cristofanelli et al.. Their study examines how social immaturity, self-representation issues, and thought process challenges contribute to emotional dysregulation. By shedding light on these dynamics, the research provides critical insights for developing targeted interventions that support emotional health during this pivotal stage of development.

The protective role of purpose in life— a key component of eudaimonic wellbeing— is explored by Barcaccia et al., who link it to reduced depressive symptoms and enhanced resilience in adolescents. Their findings emphasize that purpose in life can foster emotional regulation as part of identity formation, highlighting its critical role in navigating the challenges of adolescence.

Other contributions highlight the influence of external factors on emotional regulation. Ríos-Rodríguez et al. review the benefits of contact with nature, emphasizing its potential to reduce stress and enhance emotional wellbeing. Sansone extends this discussion by focusing on the role of mindful parenting and secure attachment in fostering emotional health. Her work underscores the relational and embodied nature of emotional regulation, situating it within the broader context of societal and community support.

The complexities of emotional regulation in the context of trauma are explored by Rojas-Saffie et al.. Their interdisciplinary study examines the symptomatology of post-traumatic stress disorder (PTSD), traditionally viewed as a form of emotional dysregulation. Drawing on Thomistic anthropology, they explore the adaptive potential of PTSD symptoms as mechanisms of regulation. While the authors ultimately conclude that PTSD symptomatology aligns more closely with dysregulation, their nuanced approach highlights the interplay between voluntary and involuntary emotional processes, offering a foundation for rethinking therapeutic interventions.

Similarly, De Vincenzo et al. investigate emotional regulation in the context of chronic pain. Their study of patients with autoimmune inflammatory rheumatic diseases emphasizes the role of cognitive reappraisal and experiential avoidance in maintaining wellbeing despite pain. By demonstrating how psychological flexibility can mitigate the impact of pain on emotional wellbeing, the authors provide a roadmap for integrating emotional regulation into pain management strategies, paving the way for innovative clinical applications.

Philosophical dialogues further enrich this Research Topic. Rojas-Saffie and García-Matte explore emotional self-regulation through Thomistic anthropology, offering a nuanced perspective that integrates reason, virtue, and emotional habits. Their interdisciplinary approach bridges psychological and philosophical insights, contributing to a deeper understanding of how self-regulation shapes personality and ethical behavior.

Taken together, these contributions reveal the multifaceted nature of emotional regulation as a contributor to flourishing. There is no flourishing without emotional (or affective) regulation, and emotional regulation does not reach its deepest meaning unless it is directed toward a flourishing, fulfilled life (Valenzuela, 2022, 2024). This Research Topic emphasizes the integrative potential of emotional regulation, encompassing cognitive, affective, and social dimensions. By addressing the interplay between motive, value, and action, the research presented in this journal issue advances our understanding of how emotions shape individual and collective wellbeing.

The practical implications of this work are significant, offering actionable insights for education, therapy, and policy. From developing mindfulness-based programs to designing interventions for trauma survivors, the studies in this Research Topic demonstrate the transformative potential of emotion regulation strategies. By embracing the complexity of emotions, researchers and practitioners can foster a more comprehensive approach to wellbeing, one that recognizes the cultural, developmental, and ethical dimensions of human flourishing (Fowers et al., 2024).

Emotion regulation is not merely a cognitive process but a fundamental aspect of what it means to live well. By integrating perspectives from diverse disciplines, this Research Topic paves the way for a richer understanding of how emotions influence our lives. It is our hope that these contributions will inspire further exploration, deepening our appreciation of the role of emotions in fostering individual and societal flourishing.
